# The impact of a nurse mentoring program on the quality of labour and delivery care at primary health care facilities in Bihar, India

**DOI:** 10.1136/bmjgh-2019-001767

**Published:** 2019-12-18

**Authors:** Saifuddin Ahmed, Swati Srivastava, Nicole Warren, Kaveri Mayra, Madhavi Misra, Tanmay Mahapatra, K D Rao

**Affiliations:** 1 Population, Family and Reproductive Health, Johns Hopkins University, Baltimore, Maryland, USA; 2 Department of International Health, Johns Hopkins University Bloomberg School of Public Health, Baltimore, Maryland, USA; 3 Johns Hopkins University School of Nursing, Baltimore, Maryland, USA; 4 Oxford Policy Management, New Delhi, India; 5 CARE India, Patna, India

**Keywords:** maternal health, health systems, health systems evaluation

## Abstract

**Introduction:**

Although the number of women who deliver with a skilled birth attendant in India has almost doubled between 2006 and 2016, the country still has the second highest number of maternal deaths and the highest number of neonatal deaths globally. This study examines the impact of a nurse mentoring programme intended to improve the quality of intrapartum care at primary healthcare centre (PHC) facilities in Bihar, India.

**Method:**

We conducted an evaluation study in 319 public PHCs in Bihar, where nurses participated in a mentoring programme. Using a quasi-experimental trial design, we compared the intrapartum quality of care between the mentored (n=179) and non-mentored PHCs (n=80). Based on direct observation of 847 women, we examined percent differences in 39 labour, delivery and postpartum care-related recommended tasks on five domains: vital sign and labour progress monitoring after admission, second and third stages of labour management, postpartum counselling, infection prevention and essential newborn care practices.

**Results:**

A significantly higher proportion of women at mentored PHCs received the recommended clinical care, compared with women at non-mentored PHCs. The overall total score of quality of care, expressed in percent of tasks performed, was 30.2% (95% CI: 28.3 to 32.2) in the control PHCs, suggesting that less than one-third of the expected tasks during labour and delivery were performed by nurses in these facilities; the score was 44.2% (95% CI: 42.1 to 46.4) among the facilities where the nurses were trained within last 3 months. The task completion score was slightly attenuated when observed 1 year after mentoring (score 39.1% [37.7–40.5]).

**Conclusion:**

Mentoring improved intrapartum care by nurses at PHCs in Bihar. However, less than half of the recommended normal delivery intrapartum tasks were completed by the nurse providers. This suggests the need for further improvement in the provision of quality of intrapartum care when risks to maternal and perinatal mortality are highest.

**Key questions**

**What is already known?**

- There is significant knowledge gap on the effects of mentoring on improving skills and competency of nurses and midwives.
- The results of limited numbers of studies on the mentored training of nurses were mixed.

**What are the new findings?**

- This study utilised a quasi-experimental, posttest only non-equivalent control trial design to evaluate the effectiveness of a mentor training programme of nurses at primary healthcare centres in Bihar, India.
- The study shows improving the quality of labour and delivery management and reduction of harmful practices is possible with nurse mentoring but sustainability is challenging.

**What do the new findings imply?**

- Although mentoring may improve skills and competency of health workers, the intervention is not sufficient to ensure high-quality recommended care during labour and delivery.
- Many good practices regress in the absence of ongoing mentoring; there are critical needs to improve and sustain good practices.

## Introduction

Nearly all maternal deaths in low-income and middle-income countries are considered preventable.^1^ Increasing the number of facility-based deliveries attended by a skilled birth attendant (SBA) is a key safe motherhood intervention strategy for reducing maternal mortality.^2 3^ The rationale is that most maternal complications are preventable but not predictable,^4^ and most maternal deaths occur during delivery and the immediate postpartum period. It is expected that facility-based deliveries with an SBA would ensure immediate access or referral to emergency obstetric care if any complication arises. The proportion of women attended at birth by an SBA, a key indicator for monitoring progress towards the third Sustainability Development Goal (SDG 3.1.2) to reduce global maternal mortality, has increased from 61% in 2000 to 78% in 2016. Nevertheless, in developing countries, the maternal mortality ratio remains at greater than that was observed in developed countries in the 1930s.^5^ Moreover, almost half of the global 2.6 million stillbirths occur during labour and delivery in spite of high SBA coverage and deliveries at health facilities.^6^ High maternal and perinatal deaths of newborns at health facilities are attributed to poor quality of intrapartum care and inadequate services, and there is a consensus that quality of care should be monitored and improved.^7 8^ A recent study suggests that half of all maternal deaths and 1 million neonatal deaths could be prevented by ensuring high quality of healthcare delivery system.^9^

India has made major efforts towards increasing institutional deliveries in recent years. Since the launch of the Suraksha Yojana^10–12^ and National Rural Health Mission programme in 2005,^13–15^ institutional births have increased substantially from 39% to 79% in 2015–2016.^16^ Yet recent studies show that increases in institutional deliveries do not reflect the expected reductions in maternal and infant mortality, which also require improvement in quality of care at institutions.^17^ About 45 000 maternal deaths occurred in 2015 in India, second only to Nigeria.^18^ In 2016, maternal conditions accounted for nearly 6.4% of all deaths in women of reproductive age, primarily from haemorrhage, sepsis and other infections, abortions, miscarriage and ectopic pregnancies, maternal hypertensive disorders and others.^19^ The highest number of neonatal deaths in the world occurs in India—750 000 annually.^20^ Neonatal disorders such as preterm birth complications, birth asphyxia and trauma, haemolytic disease and jaundice, neonatal sepsis and others account for 44% of all under-5 deaths.^21^ Neonatal mortality rate in India (26 per 1000 births) is much higher than the global rate (19 neonatal deaths per 1000 live births).

Assessments of maternity care in health facilities in India indicate poor overall quality of care, with high variability across states and within districts of the same states.^22–24^ Access to knowledgeable and highly skilled providers increases with higher socioeconomic status;^25^ and quality indicators are better in private versus public facilities.^23 26^ National and state governments have sought to improve the quality of care for maternal and infant health services, most recently through the National Health Mission, by improving infrastructure, supervision, staff training and supplies. National policy guidelines have also increasingly highlighted person-centred maternal care focusing on quality. However, clinical competence of health workers remains poor, leading to poor identification and management of labour and its complications.^26 27^ In addition to labour care, nursing and midwifery staff are also responsible for outreach activities and the collection and documentation of facility service indicators. This leads to both over-stretched staff and poor delivery outcomes, which further lowers demand for maternal health services in public facilities.^27 28^ Some harmful delivery practices are also widespread in the Indian context including but not limited to routine labour augmentation with uterotonics, application of fundal pressure, non-evidenced-based patterns and practices of maternal and fetal intrapartum monitoring, harmful traditional cord care and poor infection control and discharge.^29–33^

Maternal health providers in Bihar and other states in India have identified the availability of adequate health infrastructure, functioning HMIS and evidence-based training with supportive supervision as enablers of quality in maternal health service provision.^30 34^ However, intervention studies on improving nurse providers skills and practices have used varied modes of training and duration, with some studies noting significant improvements in adherence to evidence-based practices and reduction in harmful practices like labour augmentation, episiotomy for primigravida and application of fundal pressure.^35–38^ Studies reported mixed results on other practices, such as position of mother during delivery, the use of partograph and hand-washing.^36^ One study reported that while training resulted in more than 90% adherence to WHO’s Safe Birth Checklist of essential birth practices, these practices decreased when the trainer was absent.^37^ Another study found reported improvements in nurse skills but no significant improvements in practices such as diagnosis and management of complications.^39^ Two studies from Bihar found improvements in overall essential practices for the mother and neonate immediately posttraining, which declined 1-year postintervention but remained above baseline levels.^35 40^ Another study found that while adherence to the WHO’s Safe Birth Checklist improved among intervention versus control providers, this was not associated with improved birth outcomes such as perinatal or maternal deaths or severe maternal complications.^41^ Many studies also highlight the importance of adequate human resources, monitoring and supporting supervision and infrastructure and supply chains in sustaining training gains.^36 42 43^

This paper examines the effect of a nurse mentoring intervention on improving the quality of delivery care provided in public primary healthcare centres (PHC) in Bihar, India.

## Methods

The study is based on data from an independent evaluation of the AMANAT nurse mentoring programme, which is described below.

### Nurse mentoring program in Bihar: AMANAT

The Government of Bihar (GoB), the Bill and Melinda Gates Foundation and CARE India initiated the *Integrated Family Health Initiative* (IFHI) programme in 2011 to decrease mortality and improve malnutrition of mothers and infants in selected districts of Bihar, which is the third largest state in India by population size and one of the poorest among 29 states.^44^ The state’s maternal mortality ratio is 208 deaths per 100 000 livebirths and neonatal mortality rate of 36.7 deaths per 1000 live births, which are higher than the national rates.^45^ About 64% of births occur in facilities in Bihar, compared with 78.9% nationally. A key objective of the IFHI programme was to improve the quality of key family health and delivery services. As part of this programme, a Nurse Mentoring training was implemented to improve the quality of delivery care^46^ by mentoring nurses at facilities offering basic and comprehensive emergency obstetrics and neonatal care.^47^ CARE India implemented this mentoring programme, known as AMANAT. The programme was described earlier.^48^

The AMANAT training curricula included modules on basic nursing procedures, infection prevention, care during normal labour and immediate postpartum periods, management of complications including haemorrhage and birth asphyxia. Content on documentation, reporting, team building and communication were also included. The training methodology included lectures, bed-side mentoring, live-debrief, demonstration, clinical practice and structured simulations incorporating birth-simulator Mama Natalie, Neo Natalie and a video-based PRONTO simulation Pack.^49^ Simulations included both normal and complicated delivery including team-based communication exercises. The AMANAT programme had approximately 20 teams of mentors; each with two nurse mentors and one master nurse mentor. Each team of mentors trained a group of approximately 6–8 nurses each in four PHCs in 1 month; the mentoring team would spend 1 week in each PHC before moving to the next. Each of the four phases of AMANAT mentoring were 7–9 months long; therefore, every PHC was exposed to 7–9 weeks of direct mentoring. The duration of each phase was flexible and varied depending on the time needed to cover the curricula and mentor’s assessment of mentees progress. The nurse mentors with a minimum education of BSc degree were hired by the CARE India, and they were recruited from across the states of India. The CARE India provided additional training to the mentors on team building, communication skills and debriefing skills.

Oxford Policy Management, India, and Johns Hopkins University undertook an evaluation study in 2016 to examine the impact of the AMANAT Nurse Mentoring programme on process indicators for quality of nurse practices for delivery care in PHCs in Bihar, India. The AMANAT programme was implemented in phases, which provided the evaluation team opportunities to examine both the immediate and longer term impact of the programme on nurse delivery practices. The selection of intervention health facilities and nurses to be trained within them was determined by CARE India based on need and capacity criteria. Several criteria determined selection of a PHC: availability of nurses at the PHCs, the volume of deliveries, the infrastructural readiness of the PHC, the willingness of the PHC management to undertake mentoring and the proximity of the PHC to other PHCs in the area such that mobile mentoring teams could easily rotate between them. In our analysis, we include many of these selection criteria for matching with the control health facilities using counterfactual propensity score matching. Facilities were selected in the intervention pilot and four consecutive phases, with 80 facilities in each phase.

The evaluation team adopted a quasi-experimental, post-test only non-equivalent control trial design. Non-mentored PHCs were selected as the control group. We describe below the selection procedure of the PHC facilities in the AMANAT programme.

#### Selection of facilities

There are 534 block-level (subdistrict administrative unit) health facilities in Bihar. The AMANAT programme was implemented in four phases, such that, in each phase 80 PHCs were purposively selected. Further, in addition to the 320 PHCs that were exposed during the AMANAT programme (2015–2016), in an earlier pilot (Ananya) phase, another 80 PHCs were exposed to mentoring (2011–2013). Therefore, out of 534 PHCs, around 134 PHCs were not exposed to mentoring. This evaluation focuses on the 240 PHCs in phases II, III and IV. We exclude PHCs in the pilot (Ananya) phase, as well as, phase I PHCs because they had completed mentoring about 2 years prior to the survey date and we expected significant number of transfers of mentored nurses during this time. The selected PHCs were first observed twice via repeat cross-sectional surveys—first, just after mentoring was completed and a second survey some months afterwards, depending on the phase.

The number of facilities surveyed in the evaluation study are presented in Web online supplementary appendix 1. The 240 PHCs in phases II, III and IV of the AMANAT programme served as our sampling frame. For phases II and III, which began mentoring at approximately the same time, 40 PHCs from each phase were randomly sampled and a total of 79 out of these 80 selected PHCs were surveyed (one facility in phases II and III received the intervention in the initial pilot phase between January 2011 and December 2013 and was excluded). From phase V, 80 PHCS were selected and all were surveyed. Thus, a total of 159 mentored PHCs constitute the pool of intervention group PHCs. Data were collected from facilities at a minimum of two time points: within 3 months of completing the mentoring programme, and 1 year after completion.

10.1136/bmjgh-2019-001767.supp1 Supplementary data

From 134 non-mentored facilities, 80 PHCs were selected as comparison (control) group from adjacent blocks of mentored facilities as follows. Of the 134 health facilities that were not mentored under AMANAT, seven facilities reported 15 or fewer deliveries per month in the state Health Management Information System (HMIS) and were excluded. The remaining 127 facilities were mapped to assess geographical proximity to the selected mentored facilities. From these PHCs, 80 PHCs that were closest to a mentored PHC, that is, from adjacent or nearest block as an intervention PHC, were purposively selected.

#### Selection of deliveries for quality of care observation

Following approval from the GoBihar and the Medical Officer in-charge of the PHC, trained nurse enumerators observed a convenience sample of deliveries over a 4-day period (excluding nights). Nurses provided written consent. Due to low literacy level and cultural considerations, labouring women provided oral consent.

### Data collection

A structured checklist was used to collect information on nurse delivery practices based on AMANAT training curricula and GoB and Indian care guidelines. The checklist included items on initial assessment, second and third stages of labour, fourth stage of labour and postpartum observation, respectful maternity care, complications and referrals. CARE India, the Bill and Melinda Gates Foundation and AMANAT implementers were involved in the development of the checklist.

Deliveries were directly observed by pairs of trained nurse enumerators in each facility. Nurse enumerators observed delivery care and provided no clinical support. Information on deliveries, complications, referrals, was extracted from health facility registers of all health facilities included in the study. Data were collected continuously in phases corresponding to intervention phases from July 2016 to October 2017.

Nurse enumerators were required to have at least bachelors level nursing qualification. Enumerators participated in an initial 2 week training on the use of the checklist with periodic week-long refresher trainings every 6 months. None of the data collectors were involved in providing mentorship at any stage in the programme. Data collection supervisors oversaw incoming data; 10% of all entered data was back-checked with original checklists.

#### Facility assessment

A structured questionnaire was used to collect information on facility characteristics such as accessibility, labour room and maternity wards bed capacity, power supply, running water and handwashing station availability, availability of different diagnostic tests and medicines, human resources and delivery load among others. These variables were matched for mentored and non-mentored facilities.

### Analytical plan

Our assessment framework was based on national and international guidelines^50 51^ and comprised indicators for obstetric, fetal and neonatal care including women’s initial assessment; management of the second, third, and fourth (postpartum) stages of labour. These included management of labour and birth and essential new-born care and appropriate infection control practices.

Observed items were coded 1 if a particular action was performed and 0 if not. Items were aggregated at the level of each obstetric or fetal/neonatal clinical practice domain, stage of delivery and finally as an overall practice score. The scores were calculated for each observation domain and for overall total items. The scores are expressed as percent of recommended essential tasks completed by the nurses. Descriptive statistics were produced for both study groups.

While randomised controlled trials are the gold-standard for evaluating interventions, AMANAT was a scaling-up of the Ananya programme, targeting low-performing areas with poor health indicators in a stepwise manner to eventually cover the whole state. We therefore designed the evaluation as a quasi-experimental trial. Our initial analyses suggest some differences between the intervention arms, and we matched control PHCs to the mentored PHCs on selected characteristics using the coarsened exact matching (CEM) method.^52 53^

Facilities were matched on tracer facility indicators selected from the WHO Service Availability and Readiness Assessment framework,^54^ such as service utilisation (mean number of monthly deliveries in the past year), basic amenities (continuous power supply; handwashing station in labour room), infection prevention and essential medicines (oxytocin) availability. Connectivity to a paved road was a measure of access and a local developmental indicator. These variables were modelled to improve covariate imbalance. A summary of matching variables before and after the two sets of matching is shown in table 1. The results suggest that matching worked well. A disadvantage of this method was that facilities that could not be matched were excluded, reducing the sample size.

**Table 1:** Characteristics of matched and unmatched sample facilities

Indicator	Sampled PHC facilities		Matched PHC facilities*	
	Mentored	Comparison	Mentored	Comparison
N (PHC)	**159**	**80**	**133**	**56**
Connected to paved road (%)	90.6	85.0	91.8	91.8
Electricity always available (%)	85.5	87.5	90.3	90.3
Labour room has functional handwashing	94.3	91.3	100.0	100.0
Oxytocin available	79.9	77.5	82.8	82.8
Number of hospital beds (mean)	12.2	21.6	10.9	11.1
Number of deliveries in past year (mean)	192.9	126.3	193.3	186.8

*Weighted for coarsened exact matching between treatment and control groups.

PHC, primary healthcare centre.

Although the study collected data from 1022 women, 175 cases were dropped when the study was limited to CEM matched facilities. The study is limited to data from matched facilities (n=189) and direct observations of 847 women at different stages of labour at mentored and non-mentored facilities (383 women were observed at admission, and 813 women were observed during second and third stages of labour, of whom 349 cases overlapped—observed at admission and during the second and third stages of labour and others were not observed at the time of admission but enrolled before the starts of the second stage of labour). All tasks could not be observed for all delivering women due to the non-availability of the observing nurses at the time of admission or during the labour progression or delivery, or due to referral of delivering women to other facilities.

Analyses were weighted for imbalance between mentored and non-mentored PHCs and adjusted for clustering effects (design-effects>1) due to multiple observations of deliveries at facilities. We examined percent differences in clinical care-related tasks between mentored and non-mentored facilities by Rao-Scott second-order corrected χ^2^ test. We generated a set of summary scores from 0 to 100 on five care domains: vital sign and labour progress monitoring, management of second and third stages of labour, postpartum care and counselling, infection prevention and essential newborn care practices. Scores were generated for each delivery based on number of items observed and number of items completed. The scores are expressed as percent of items completed. The use of uterotonic drugs after birth was measured at two time points: within 1 and 3 min. The use within 3 min was excluded from the score construction. To account for clustered data, differences in scores between mentored and comparison facilities were compared with adjusted Wald test.

### Patient and public involvement

This is a cross-sectional observational study that collected data by observing quality of care received by women from nurses during their labour and delivery. No patient or public was involved in the design or survey instruments development of the study. No individual level data were collected, and the study subjects were not identifiable for sharing the study results.

## Results

### Vital sign and labour progress monitoring at admission

Vital signs (blood pressure, pulse and temperature) monitoring at admission was low in the non-mentored facilities, but higher among women admitted at mentored PHCs at both survey rounds (table 2). Haemoglobin and urine testing were low at all PHCs studied. None of the non-mentored PHCs used partographs to monitor labour progress, which is recommended by the WHO since 1990s. However, partographs were used among one-third of labouring women at mentored PHCs. Abdominal examination for fundal height or contractions was significantly higher in the mentored PHCs compared with non-mentored PHCs. Similarly, fetal heart monitoring at mentored PHCs was significantly higher, irrespective of the timing of observations—within 3 months (61.4%) or after 1 year of the completion of mentoring programme (68.8%), compared with non-mentored facilities (39.7%).

**Table 2:** Vital sign and labour progress monitoring observed on admission at mentored and non-mentored PHCs

	N	Control	N	Mentored: <3 months	N	Mentored: 1+year	P value
Temperature checked	44	0.0	154	7.1	184	4.9	0.2907
Pulse checked	44	14.5	154	35.7	184	34.4	0.1053
Blood pressure checked	45	41.1	154	46.1	185	65.8	0.0184
Vaginal examination conducted	44	96.2	154	46.8	184	93.5	<0.001
Urine test performed	44	0.0	154	0.6	184	0.5	0.8700
Haemoglobin test conducted	44	4.0	154	1.3	184	3.3	0.4984
Partograph initiated	44	0.0	195	32.8	236	34.9	0.0112
Abdominal examination conducted	44	18.1	200	57.0	251	62.3	0.0003
Fetal heart rate monitored	47	39.7	197	61.4	219	68.8	0.0417

Some items were not observed for all subjects. Sample size may vary by observation items.

### Management of second and third stages of labour

Vital signs monitoring was very low for women in the second stage of labour in mentored and non-mentored PHCs (table 3). Monitoring of uterine contractions was also very low in both mentored and non-mentored PHCs.

**Table 3:** Labour progress monitoring and management of second and third stages of labour at mentored and non-mentored PHCs

	N	Control	N	Mentored: <3 months	N	Mentored: 1+year	P value
Contractions assessed by placing hand on woman’s abdomen for 10 min	80	0.0	319	3.4	414	0.0	0.0941
Pulse assessed	80	0.1	319	6.3	414	2.9	0.0032
Temperature assessed	80	0.0	319	0.6	414	0.2	0.5942
Blood pressure assessed	80	9.6	319	10.7	414	5.8	0.1056
Support provided to perineum during delivery of head	80	40.0	319	60.2	414	49.2	0.0450
Checked for presence of second twin	80	7.5	319	7.2	414	1.5	0.0205
Uterotonic administered within 1 min of birth	80	6.5	319	43.9	414	41.4	0.0001
Uterotonic administered within 3 min of birth	80	31.9	319	76.2	414	72.9	<0.001
Fundal pressure was not applied during labour	80	54.6	319	80.9	414	84.8	0.0001

PMC, primary healthcare centre.

Perineal tears can be a complication of poorly conducted vaginal delivery. A significantly higher proportion of women received support of the perineum during delivery of head of the baby in the mentored PHCs (60%) within 3 months postmentoring, compared with non-mentored PHCs (40%). However, the practice was reduced substantially in the mentored facilities after 1 year (49%).

Prophylactic use of uterotonic drugs (oxytocin alone as a first choice), that is, active management of the third stage, is recommended by the WHO for all deliveries within 1 min of delivery of the baby and before the delivery of the placenta for the prevention of postpartum haemorrhage. About 44% and 76% of women received a uterotonic drug within 1 min and 3 min of birth, respectively, in the mentored PHCs when observed within 3 months of mentoring. In contrast, only 6% of women received a uterotonic drug within 1 min and 32% within 3 min of birth in the non-mentored PHCs. Large differences persisted in the use of uterotonic drugs immediately after birth between the mentored and non-mentored PHCs even after 1 year of mentoring completion.

Use of fundal pressure is strongly discouraged by WHO recommendations. Almost half of delivering women in non-mentored PHCs received fundal pressure, compared with less than 20% of women in the mentored PHCs at both the immediate and the late rounds of observations.

### Postpartum counselling and education

Significantly higher proportions of women received postpartum counselling about skin-to-skin contact, pain and bleeding management and expectations, immediate breastfeeding and breastfeeding practices at mentored PHCs within 3 months postmentoring (table 4). However, these practices were diminished after 12 months of mentoring.

**Table 4:** Postpartum counselling observed at mentored and non-mentored PHCs

	N	Control	N	Mentored: <3 months	N	Mentored: 1+year	P value
Mother given instructions about bleeding/cramping	79	3.9	319	18.5	412	4.6	<0.001
Mother received breast-feeding education or encouragement	79	46.7	319	66.8	412	36.3	<0.001
Mother received KMC /skin-to-skin education or encouragement	79	3.6	319	21.3	412	7.5	<0.001

KMC, Kangaroo Mother Care.

### Infection prevention practices

Glove usage for vaginal examinations and delivery was high in both mentored and non-mentored PHCs (table 5). Other infection prevention measures—handwashing and proper disposal of the placenta- by nurses during labour and delivery were observed more frequently in the mentored PHCs, especially within three postmentoring.

**Table 5:** Selected infection prevention steps during labour and delivery observed at mentored and non-mentored PHCs

	N	Control	N	Mentored: <3 months	N	Mentored: 1+year	P value
Hands washed with soap and water before vaginal examination	46	13.2	64	57.8	138	42.8	0.0201
Gloves used for vaginal examination	46	100.0	64	98.4	138	97.1	0.4837
Hands washed with soap and water after vaginal examination	46	4.5	64	34.4	138	34.1	0.0005
Hands washed with soap and water before delivery	80	21.2	150	60.0	413	41.5	<0.001
Gloves used for delivery	80	97.1	150	96.0	413	94.4	0.5916
Hands washed with soap and water after delivery	80	73.3	150	86.0	413	76.7	0.1960
Placenta disposed in a yellow coloured bin	78	27.7	318	69.5	412	54.3	0.0005
Soiled dressing materials disposed in dedicated bin	79	28.3	319	69.9	413	50.0	0.0002

Some items were not observed for all subjects. Sample size may vary by observation items.

### Essential newborn care practices

The WHO recommends late cord clamping (1—3 min afterbirth) while simultaneously initiating essential newborn care. Where neonatal resuscitation is needed, early cord clamping within 1 min is recommended. Delayed cord clamping was more common in the mentored PHCs within 3 months postmentoring (table 6). Immediate skin-to-skin care, immediate drying and wrapping the baby practices were also substantially higher in the mentored PHCs. A significantly higher proportion of newborns were checked for cord around the neck (nuchal cord) in the mentored PHCs, especially within 3 months postmentoring. Sustaining many of these practices, however, remained a challenge. When observed after 1 year after the completion of mentoring programme, the practices were more similar to the non-mentored facilities.

**Table 6:** Essential newborn care infection prevention steps observed during labour and delivery at mentored and non-mentored PHCs

	N	Control	N	Mentored: <3 months	N	Mentored: 1+year	P value
Provider checked for cord around the neck of newborn	79	9.2	318	25.5	413	9.9	<0.001
Newborn immediately placed on the mother’s abdomen after birth	79	78.6	319	93.1	413	93.9	0.0007
Newborn assessed for breathing-crying at birth	79	77.1	319	90.0	413	84.5	0.0293
Newborn dried using a clean towel or cloth after birth	79	91.3	319	95.0	413	88.6	0.0247
Newborn covered using fresh towel or cloth	79	23.0	319	55.5	413	32.3	<0.001
Cord checked for pulsation before clamping	79	16.0	319	48.9	413	26.0	<0.001
Cord clamped and cut with sterile scissor/blade after waiting for 3 min ?	76	0.1	315	19.4	412	1.2	<0.001
Newborn's eyes wiped with sterile wet gauze	79	0.0	319	15.4	413	0.5	<0.001
Skin to skin care initiated after birth	79	2.2	319	18.5	412	5.3	<0.001
Newborn weighed just after delivery	79	81.2	319	81.8	413	84.0	0.8361

### Intrapartum care scores

The differences in the summary scores in five domains examined above are shown in table 7. In addition, we provide an overall total score based on all domains. For all the observed quality of delivery care domains, summary scores were significantly higher in the mentored PHCs compared with the non-mentored PHCs within 3 months postmentoring. The scores remained significantly higher 1 year postmentoring. The only exception was for postpartum counselling.

**Table 7:** Score for the quality of normal delivery care at the mentored and non-mentored PHCs

Quality of care observation domains		Mentored	
	Control	Observed within 3 months	Observed after 1 year
Vital sign monitoring after admission	29.5 (19.4–39.6)	53.9 (46.6–61.1)	59.6 (54.6–64.6)
Labour progress monitoring and management during second and third stages of labour	14.8 (11.5–18.1)	26.6 (24.5–28.7)	23.2 (21.8–24.6)
Postpartum counselling	18.0 (10.1–26.0)	35.5 (30.9–40.2)	16.2 (13.5–18.9)
Infection prevention	47.9 (40.0–55.9)	72.1 (66.3–77.9)	63.3 (59.6–66.9)
Newborn care	38.0 (34.5–41.5)	54.3 (51.7–57.0)	42.7 (41.2–44.1)
Total	30.2 (28.3–32.2)	44.2 (42.1–46.4)	39.1 (37.7–40.5)

Some items were not observed for all subjects. Sample size may vary by items. Scores were based on observed items only.

PHC, primary healthcare centre.

**Figure 1:**
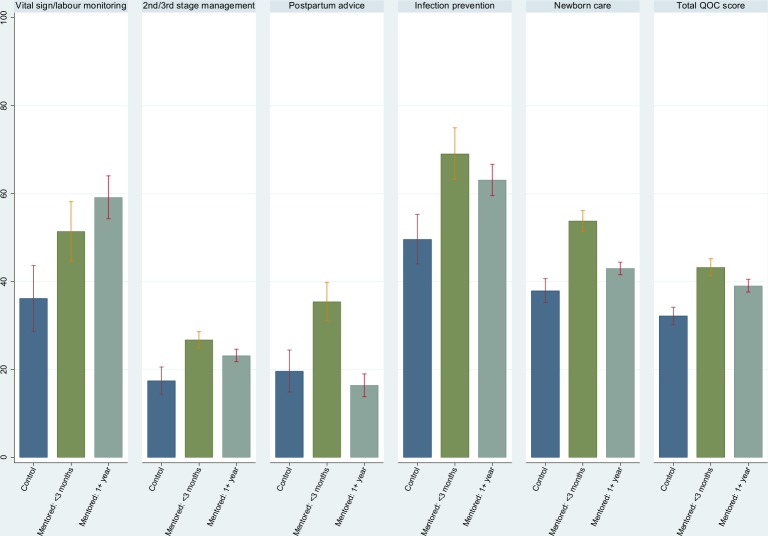
Score for the quality of normal delivery care at the mentored and non-mentored PHCs. PHC, primary healthcare centre; QoC, quality of care.

Overall, the total score was 30.2% (95% CI: 28.3 to 32.2) in the non-mentored PHCs, suggesting that less than one-third of the recommended practices examined were completed in these facilities. At 3 months postmentoring, the overall score was about 14% point higher in the mentored PHCs. Except for postpartum counselling, the mentored PHCs maintained significantly higher total scores ever after 12 month postmentoring. However, the overall score was reduced by about 5% points. The results are shown graphically in figure 1.

## Discussion

Our analyses show that women delivering at nurse-mentored PHCs in Bihar have received significantly improved quality of care, compared with women delivered at non-mentored PHCs. The quality of care remained high 1 year after the mentoring programme’s completion, though at levels that were lower compared with shortly after mentoring was completed. This suggests that the mentoring effects on improving the quality of labour and delivery care are sustainable; however, they are likely to regress if ongoing mentoring is not provided.

Across all five domains (vital sign and labour progress monitoring at admission, management of the second and third stages of labour, postpartum counselling, infection prevention, and essential newborn care practices), the overall quality scores were 14% higher in the mentored PHCs (within 3 months postmentoring) compared with matched, non-mentored PHCs. The largest gains (around 24 percentage points) were in the domains of vital signs monitoring at admission and infection prevention. This was followed by postpartum counselling (18 percentage points), infection prevention (16 percentage points) and management of second and third stage labour (12 percentage points). In other words, the greatest gains occurred in the what are arguable the least critical aspects of delivery-related care. This may reflect the persistence of poor delivery practices. For example, the main improvements in second and third stage managements were in the correct use of uterotonics and the non-application of fundal pressure; other actions such as checking temperature, blood pressure or for twins remined similarly low in the control and mentored groups.

It is important to be cautious about the extent to which quality of care can be improved by a single mentoring programme. Although the overall score was higher in the mentored PHCs (44.2% vs 30.2%), it is important to note that slightly less than half of all the recommended normal delivery intrapartum tasks were completed by the nurse providers. This strongly suggests the need for further improvements to the quality of care provided. Importantly, mentored nurses scored lowest on activities in the second and third stage labour which were foci of the mentoring programme. Moreover, the quality score in the mentored PHCs was attenuated by about 5% points after 1 year of training completed, but remained significantly higher than the non-mentored PHCs. Loss in quality scores over time was largest for postpartum counselling (19 points) and newborn care (12 points), and infection prevention (nine points), all of which were statistically significant. Changes in the management of second and third stages delivery and vital signs monitoring after admission were not statistically significant.

We recognise a number of limitations of our study. First, the study implemented a quasi-experimental trial design, introducing the potential for selection bias. We applied CEM—a statistical procedure—to match the mentored and non-mentored PHCs on a number of variables so that the facilities were as comparable as possible, except for the AMANAT intervention. Second, labour and delivery is a complex process and happens over an extended period of time, often over more than 12 hours, making observing all events and services rendered during the woman’s stay in the facility challenging. Also, observations started at different points in the delivery process, thereby leaving out practices that had already taken place. We considered these limitations in generating the quality scores, which were calculated for each woman based on the number of events/items she was actually observed for. Due to matching of facilities and because all women were not observed for all events, the number of the effective sample size was substantially reduced for a number of analyses, thereby reducing statistical power. Third, the Hawthorne effect is a known cause of bias in direct observations. Being observed may have altered the normal functioning and behaviour of the nurses at the PHCs. Fourth, often it is difficult to identify particular events correctly during labour and delivery process, for example, whether the gloves/instruments were sterilised, which may introduce misclassification bias. We excluded some infection–prevention related items and the observation of respiration monitoring because of potential misclassification bias. Fifth, only directly observed activities were included; no data from medical charts because of reliability concerns. If a practice was done and documented by the provider but not observed by the nurse enumerator, then it was not reported in our findings. Another limitation is that we have given equal weights to all delivery practices in our score construction. Some tasks are more important and critical for intrapartum care than others. However, it is difficult to assign relative weights based on importance of the task in the absence of any empirical basis. The interviewers/observers were not informed whether the PHC is mentored or non-mentored, but we recognise that it was not possible to fully blind and conceal the trial arm assignment to them. Another challenge was that nurses at public PHCs were occasionally transferred to other facilities. It is possible that some of the nurses at mentored facilities were transferred to non-mentored facilities and vice-versa. This may attenuate the effect of mentoring in our “intent-to-treat” analyses of the PHCs, especially when observed after 12 months of mentoring completed.

Nevertheless, we collected data across five domains: vital sign and labour progress monitoring at admission, management of the second and third stages of labour, postpartum counselling, infection prevention and essential newborn care practices. The overall quality scores were 14% higher in the mentored PHCs within 3 months postmentoring, compared with matched, non-mentored PHCs. However, the score slightly attenuated after 1 year, which may suggest the challenges of sustaining high quality of care after the mentoring ends. There are critical needs to understand how mentoring programme can be made more effective for improving and sustaining good practices in resource poor settings. Efforts must be undertaken to sustain quality of care through refresher training, institutionalising accountability and strict monitoring of compliance with the standardised protocol of clinical practice. Nurse mentoring has emerged as an attractive intervention for training nurses, especially for junior nurses and in low performance areas in many countries, and the lessons from study may help in addressing some of the challenges.

A number of studies in India have suggested poor quality of intrapartum care in health facilities. Along with the promotion of deliveries with a SBA, it is critical to ensure high-quality intrapartum care at all levels of health facilities. Women may bypass poor quality PHCs and overburden higher level facilities that may adversely affect treatment of women in need of emergency obstetric care at referral hospitals. Our study has identified several deficiencies in the routine tasks during labour and delivery management, which are commonly recommended by the WHO as the standard practice for improving quality of maternal and newborn care in health facilities.^46^ Completion of these tasks as per a standardised clinical practice for delivery care is essential for identifying maternal and fetal complications early, ensue prompt treatment and reduce mortality risks.

High-quality intrapartum care is important for assuring safe delivery and healthy birth outcomes, and also pivotal for improving deliveries at health facilities. Several studies suggested that perceived quality of care at health facilities affects women and families’ decision to deliver at health facilities: higher percent of women delivered at a health facility when the village rated the facilities as excellent or considered that the doctors and nurses have good skills.^55 56^ In contrast, experience of poor quality of services during previous delivery deter women from delivering at health facilities. In collaborations with developmental and health partners, India has taken a number of measures in recent years to improve and ensure high quality of care during labour and delivery. A positive childbirth experience of all women delivering at a health facility and rapid reduction of maternal and perinatal deaths can only prove its success.
