# Supplementary material for: The impact of a nurse mentoring program on the quality of labour and delivery care at primary health care facilities in Bihar, India
Source: BMJ Glob Health. 2019 Dec 18;4(6):e001767. doi: 10.1136/bmjgh-2019-001767 (PMC6936590; doi:10.1136/bmjgh-2019-001767)
Supplement: Supplementary data [file bmjgh-2019-001767supp001.pdf]

Web Appendix:

Web Table 1: Health facilities surveyed under the AMANAT program

Health facilities	Year of training	Sample size of PHC for the quasi-experimental study n/N
A. Phase 0 Ananya (66)	2011	0/66
B. Phase 1 (80)	February-October 2015	80/80
C. Phase 2-3 PHCs (79/160)	October 2015-July 2016	79/160
D. Phase 4 PHCs (80/80)	July 2016-January 2017	80/80
E. Non-mentored PHCs (80/134)	-	80/134
